# The Effect of Viewing Eccentricity on Enumeration

**DOI:** 10.1371/journal.pone.0020779

**Published:** 2011-06-10

**Authors:** Melanie Palomares, Paul R. Smith, Carole Holley Pitts, Breana M. Carter

**Affiliations:** Department of Psychology, University of South Carolina, Columbia, South Carolina, United States of America; Istituto di Neuroscienze, Italy

## Abstract

Visual acuity and contrast sensitivity progressively diminish with increasing viewing eccentricity. Here we evaluated how visual enumeration is affected by visual eccentricity, and whether subitizing capacity, the accurate enumeration of a small number (∼3) of items, decreases with more eccentric viewing. Participants enumerated gratings whose (1) stimulus size was constant across eccentricity, and (2) whose stimulus size scaled by a cortical magnification factor across eccentricity. While we found that enumeration accuracy and precision decreased with increasing eccentricity, cortical magnification scaling of size neutralized the deleterious effects of increasing eccentricity. We found that size scaling did not affect subitizing capacities, which were nearly constant across all eccentricities. We also found that size scaling modulated the variation coefficients, a normalized metric of enumeration precision, defined as the standard deviation divided by the mean response. Our results show that the inaccuracy and imprecision associated with increasing viewing eccentricity is due to limitations in spatial resolution. Moreover, our results also support the notion that the precise number system is restricted to small numerosities (represented by the subitizing limit), while the approximate number system extends across both small and large numerosities (indexed by variation coefficients) at large eccentricities.

## Introduction

Our visual abilities depend on viewing eccentricity. Visual resolution is high for objects shown at the fovea, but falls progressively in the periphery [1], [2]. Notably, viewing eccentricity effects are task-dependent. For example, motion detection [3], [4] and detection of closed contours [5], can be as good in the periphery as in the fovea, but discrimination of phase ([6]; see also [7]), mirror-symmetric objects [8] and position [9] declines precipitously. Visual crowding (e.g., [10]) and slower reading speed [11], [12], [13] in the periphery suggest that processing of multiple elements might be particularly poor with eccentric viewing. In the current study, we investigated how the apprehension of multiple elements via visual enumeration changes in viewing eccentricity. We specifically evaluated whether viewing eccentricity changes “subitization”, the rapid apprehension of small number up to 4 items [14].

Many studies have shown that quick enumeration of items is fast and accurate for small numbers [15], but slow and inaccurate for large numbers [16], [17], [18], [19], [20]. Subitization has been thought to be the embodiment of the precise number system, which has special status within small numerosities. The precise system is thought to be distinct from the approximate number system, which is often associated with large numerosities [21]. There have been several theories of subitization, including the use of indexes (in FINST) [19], [20], polygon formation [18], memory capacity [22] spatial frequency limits [16] and spatial similarities [23].

However, the idea that two separate cognitive mechanisms underlie subitization and estimation is still debated [24], [25]. Some evidence suggests that the approximate number system operates continuously across small and large numerosities in addition to the precise number system that only operates across small numerosities (4 or fewer) [26]. Enumeration within and beyond the subitizing limit is modulated by attention [26], [27], [28], [29], [30] and is susceptible to the effects of adaptation [31]. Moreover for low contrast stimuli, enumeration functions do not show a discontinuity in accuracy [32]. Together, these results show large attentional loads and high perceptual difficulty modulate that subitization.

Here, we characterized enumeration functions for constant sized stimuli (Experiment 1) and for stimuli that scaled with viewing eccentricity (Experiment 2). Does subitizing capacity and enumeration variability change with viewing eccentricity? Does changing the stimulus size in proportion to viewing eccentricity eliminate the enumeration inaccuracies and imprecision with more peripheral viewing? If simple size scaling restores enumeration functions to match data at near-fovea levels, then it would suggest that decrements in enumeration performance with eccentric viewing is due to insufficient resolution to detect individual elements. Alternatively, if size scaling does not reduce enumeration errors to near-fovea levels, then it would suggest that in addition to poor spatial resolution, peripheral vision has unique limitations in processing multiple elements. This study has implications for visual disorders such as strabismus, a misalignment of the eyes, which has been proposed to result in central vision to be functionally similar to normal peripheral vision [33], and macular degeneration, in which the visual periphery is used to see due to a central scotoma.

We aim to extend a study by Parth and Renschler [34], which used briefly presented dots organized in a linear array. In the current study, we displayed our elements along a circular path at a single eccentricity to remove a confounding cue of length differences. We also controlled for changes in brightness in our display by using grating stimuli rather than dots at a single luminance value.

## Methods

### Ethics Statement

The protocol for this study was approved by the Institutional Review Board (IRB) at the University of South Carolina. Participants signed an IRB-approved informed consent form before experiments were conducted. Participation was voluntary; course credit was given for participation.

### Participants

Thirty-four undergraduates with normal or corrected to normal vision were recruited via the psychology study participant pool at the University of South Carolina. Nineteen students participated in Experiment 1 and fifteen in Experiment 2.

### Stimuli, Apparatus and Procedure

**Figure 1:**
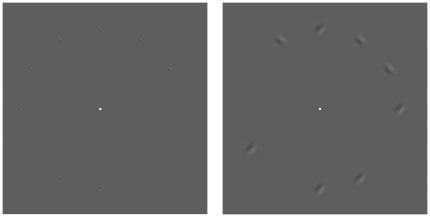
Example stimuli. Fixate on the white square and enumerate the number of gratings in the periphery. Small gratings are harder to enumerate than large gratings. Sizes were not scaled in Experiment 1 (left) but were scaled in Experiment 2 (right) according to a cortical magnification factor. There are 8 gratings in both of these panels.

The stimuli were presented on a 21″ Elo Touchscreen, using an Apple MacMini desktop computer with Matlab software featuring the Psychophysics Toolbox [35]. The stimuli were sinusoidal gratings presented along 12 possible positions on a virtual circle at viewing eccentricities of 2.25, 4.5, 6.75, 9, and 11.25 degrees on a gray background (∼42 cd/m^2^). The minimum distance, target-center to target-center, between gratings at each eccentricity was 1.18, 2.36, 3.54, 4.71, and 5.89 deg respectively (Figure 1). The minimum inter-target distance is sufficiently large to be outside of spatial extent of visual crowding, which is about half of viewing eccentricity [10]. In Experiment 1, the stimuli were 2 c/deg gratings with a 0.5 deg Gaussian envelope. In Experiment 2, the spatial frequencies of the gratings were 2.00, 0.86, 0.68, 0.55 and 0.47 c/deg, which corresponded to 0.50, 1.15, 1.48, 1.80 and 2.14 deg in size, the 1/e radius of the circular Gaussian envelope. The stimuli scaled with viewing eccentricity using a magnification factor, M [34]

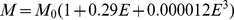
(1)E represents eccentricity and M_0_ represents the size of the stimuli at the smallest eccentricity used, 2.25 deg in this case. Although we presented our stimuli along a circular array, we used a cortical magnification estimate for the temporal visual field for simplicity. In both experiments, the gratings were randomly oriented at −45 or +45 deg and had a Michelson contrast of 54%. Observers sat 57 cm away from the screen and binocularly viewed the stimuli for 50 ms. The task was to enumerate the gratings that were presented on the screen: 0, 1, 2, 3, 4, 5, 6, 7, 8 or 9. Responses were typed on the keyboard. Correct answers were rewarded with a short beep. Because of a response bias in which observers disproportionately choose ordinal extremes, only data for 1–8 gratings were analyzed. For Experiments 1–2, each observer was given 750 trials (50 trials per block, 3 blocks per eccentricity, 5 eccentricities). Observers performed 10 practice trials at 3.75 deg of viewing eccentricity at 200 ms presentation before the actual trials.

## Results

To assess the effects of viewing eccentricity in enumeration, we evaluated accuracy and variance of enumeration. In Experiment 1, element size did not vary with eccentricity, and in Experiment 2, element size scaled with eccentricity. For stimuli with constant size in Experiment 1, the data show that enumeration becomes more error prone with increasing eccentricity. However, a subitizing capacity of about 2–4 items was generally preserved. For stimuli that were scaled in size in Experiment 2, enumeration functions were nearly identical across eccentricities, suggesting that visual enumeration in the periphery is principally limited by spatial resolution.

### Overall performance

**Figure 2:**
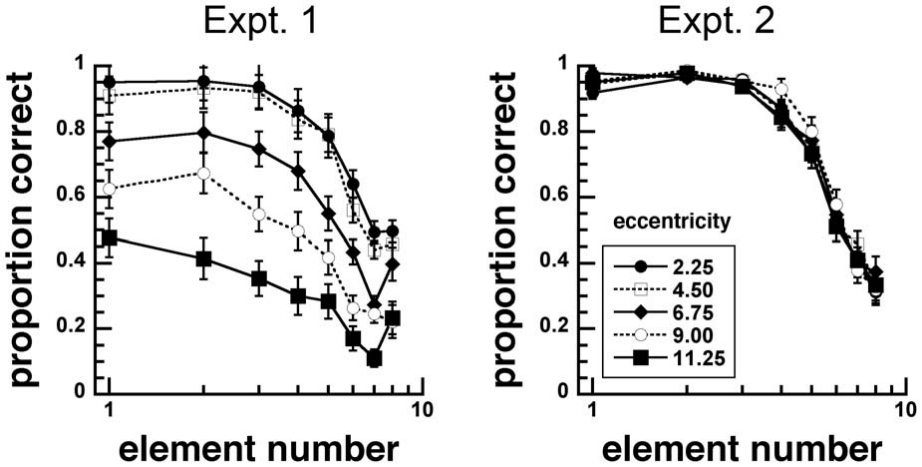
Proportion correct as a function of element number for stimuli with unscaled (Experiment 1) and scaled (Experiment 2) sizes. Enumeration errors increased as viewing eccentricity increased in Experiment 1, but not in Experiment 2.

Proportion correct was plotted (Figure 2) as a function of eccentricity. For each experiment, we conducted simple planned comparisons of proportion correct at each number to the proportion correct at 1 grating. Subitizing capacity was defined to be the largest element number at which proportions correct did not significantly deviate from the proportions correct at 1 element. We noted subitizing capacities across viewing eccentricity to determine whether the index of the precise number system is mutable.

In Experiment 1, planned comparisons indicate that subitizing capacities were, 3, 3, 3, 4 and 2 elements at 2.25, 4.50, 6.75, 9.00 and 11.25 deg eccentricities, respectively. Proportions correct at 1 element were significantly different from proportions correct at 4 elements at eccentricities of 2.25, 4.50 and 6.75 deg (p-values<0.01), while proportions correct at 5 and 3 elements were significantly different at 9.00 and 11.25 deg, respectively. We also carried out an 8 (number) by 5 (eccentricity) within-subjects ANOVAs. There were significant main effects of number, F(7, 126) = 104.216, p<0.001, and eccentricity, F(4, 72) = 66.127, p<0.001, and interaction between them, F(28, 504) = 3.870, p<0.001. These results suggest that the subitizing capacity hovered around 3 elements across all eccentricities, but that increasing viewing eccentricity decreased enumeration accuracies.

**Figure 3:**
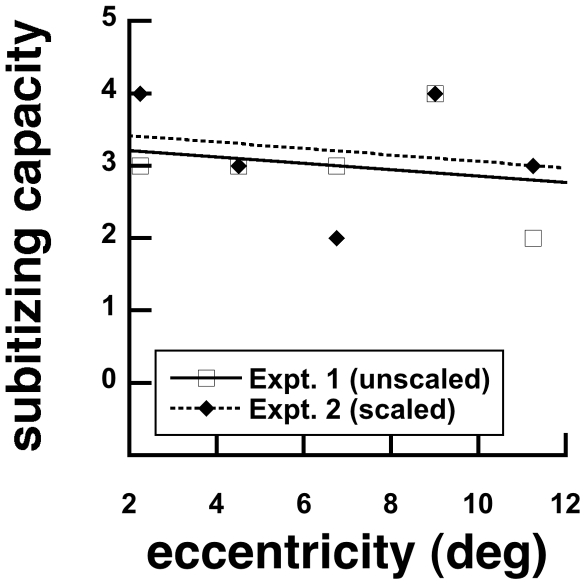
Subitizing capacities as a function of viewing eccentricity were generally flat for unscaled (Experiment 1) and scaled (Experiment 2) stimulus sizes.

In Experiment 2, subitizing capacities were 4, 3, 2, 4, 3 elements at 2.25, 4.50, 6.75, 9.00 and 11.25 deg eccentricities, respectively. An 8 (number) by 5 (eccentricity) within-subjects ANOVAs show no significant main effects of number, F(7,98) = 152.31, p<0.001. However, there was neither a main effect of eccentricity, F(4, 56) = 0.899, p = 0.471, nor interaction between them, F(28, 392) = 0.884, p = 0.639. These findings indicate that scaling the size of elements with eccentricity collapses the data into a single enumeration function. Together, results from Experiments 1–2 show that the subitizing capacities are robust between 2 and 4 elements across viewing eccentricities (Figure 3). Moreover they show that numerosity judgments in eccentric vision are limited by spatial resolution.

### Mean responses

**Figure 4:**
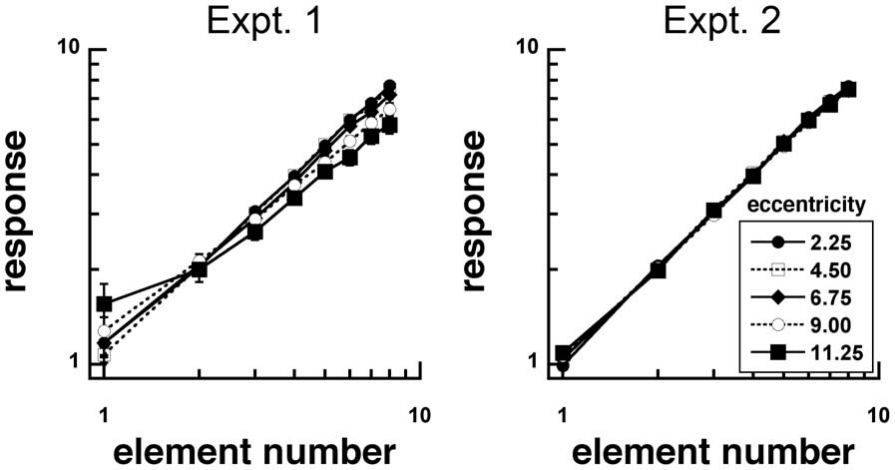
Mean response as a function of element number for stimuli with unscaled (Experiment 1) and scaled (Experiment 2) sizes. Response accuracy decreased as viewing eccentricity increased in Experiment 1, but not in Experiment 2. Response accuracy also decreased with increasing element number.

To see how enumeration accuracy, precision and variability changes with contrast, we also evaluated the mean and standard deviation of the responses as a function of number of gratings for each observer. We plotted mean responses as a function of number across five eccentricities (Figure 4) for the two Experiments.

**Figure 5:**
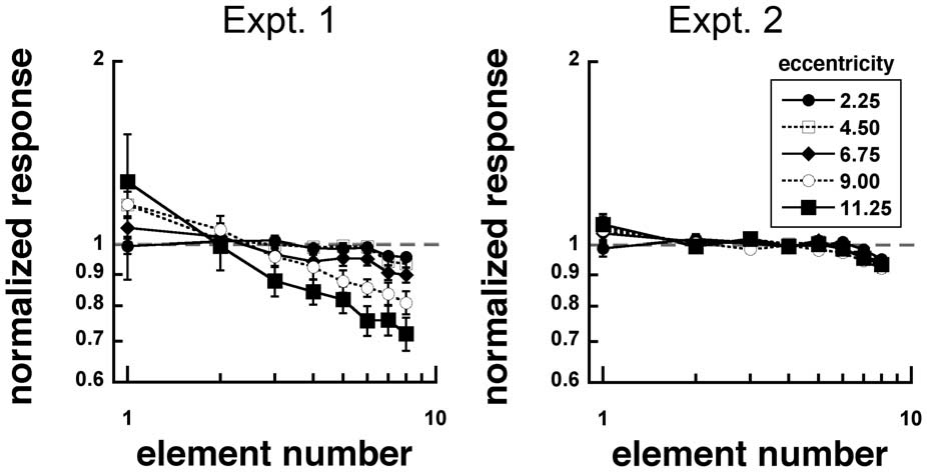
Mean normalized response (mean response/element number) as a function of element number for stimuli with unscaled (Experiment 1) and scaled (Experiment 2) sizes. Deviation from 1 (dashed line) represents inaccurate responses. Above 1 represents overestimation while below 1 represents underestimation.

In Experiment 1, mean responses generally followed element numerosity, and slopes were close to the unit slope of 1 (from 0.92 to 0.67 in log-log coordinates). These responses showed systematic deviations, decreasing in slope with increasing eccentricity. We also plotted responses against element number as a normalized ratio (response/element number) for transparency (Figure 5). If the mean response were equivalent to the actual element number, there would be ratio of 1 (dashed line). Above 1 represents overestimation, while below 1 represents underestimation. We conducted an 8 (number) by 5 (eccentricity within subjects ANOVA on log normalized responses. There were significant main effects of number, F(7, 126) = 11.796, p<0.001, and eccentricity, F(4,72) = 8.351, p<0.001, and interaction between them, F(28, 504) = 3.256, p<0.001. With increasing viewing eccentricity, observers tended to overestimate numerosities below 2 and underestimate numerosities above 2.

In Experiment 2, we conducted the same analyses. Responses as a function of element number had slopes that ranged from 0.98 to 0.94 in log-log coordinates. An 8 (number) by 5 (eccentricity) within subjects ANOVA on log normalized responses showed that there were significant main effects of number, F(7, 98) = 13.921, p = <0.001, but no effect of eccentricity, F(4, 56) = 0.987, p = 0.422, or interaction between them, F(28, 392) = 1.024, p = 0.434. Normalized responses (Figure 5) exhibited tendencies to overestimate numerosities at 1 element and underestimate numerosities at 8 elements.

The pattern of underestimation and overestimation across both experiments echo the results from a previous study on the effects of luminance contrast and enumeration [32], in which observers' responses tended to peak between 2 or 3 gratings in the low contrast conditions. The trend is similar in the current study. The mean of the response distribution is 4.45, 4.40, 4.24, 3.98 and 3.65 in Expt. 1 and 4.47, 4,44, 4,42, 4,36 and 4,42 in Expt. 2 at 2.25, 4.50, 6.75, 9.00 and 11.25 deg eccentricities, respectively. These data suggest that decreasing spatial resolution of the stimuli decreased the central tendency of observer responses from the veridical mean of 4.5 elements.

Notably, mean responses at 0 elements in Expt. 1 (not shown in Figure 5) were, 0.20, 0.19, 0.43, 0.66 and 0.92 at 2.25, 4.50, 6.75, 9.00 and 11.25 deg eccentricities, respectively. Thus, responses at 0 elements also exhibited an overestimation that increased with eccentricity: However accuracies were generally high: 0.94, 0.94, 0.88, 0.82 and 0.74 proportions correct at 2.25, 4.50, 6.75, 9.00 and 11.25 deg eccentricities, respectively. This trend suggests that the overestimation at low numerosities was not due to a perception of more numerous items, per se, but due to a few responses that land on high numerosities.

### Response variability

Variability is a metric that can assess the characteristics of the approximate number system, and has been shown to follow the rules of detection probability. Specifically, as more elements are presented, response variability is predicted to decrease as a function of the square root of the number of presented elements [32], [36].

**Figure 6:**
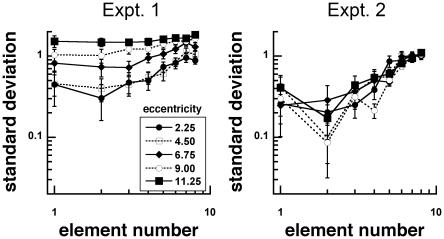
Mean standard deviation as a function of element number for stimuli with unscaled (Experiment 1) and scaled (Experiment 2) sizes. Response precision decreased as viewing eccentricity increased in Experiment 1, but not in Experiment 2. Response precision also decreased with increasing element number.

**Figure 7:**
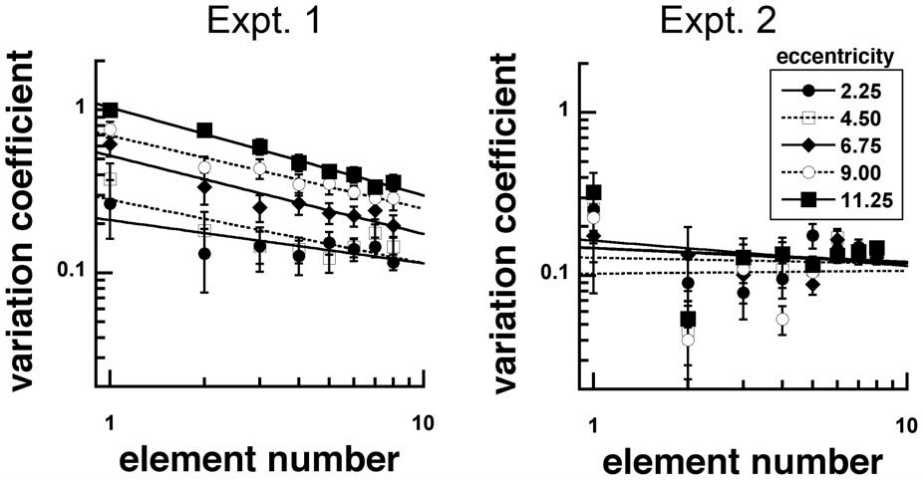
Mean variation coefficients (standard deviation/response) as a function of element number for stimuli with unscaled (Experiment 1) and scaled (Experiment 2) sizes. Variation coefficients increased as viewing eccentricity increased in Experiment 1, but not in Experiment 2.

**Figure 8:**
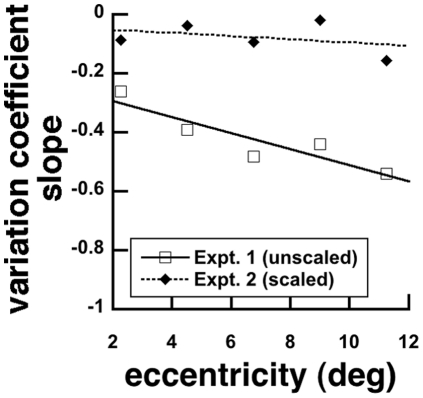
Slopes of the variation coefficients as a function of viewing eccentricity became steeper, approaching −0.5, for unscaled stimulus size (Experiment 1), but were generally flat for scaled stimulus size (Experiment 2).

Standard deviations of the responses were calculated for each observer and the average standard deviation was plotted against the number of gratings (Figure 6). The ratio of the standard deviation and mean of the responses, the coefficient of variation, was computed to determine whether variability scales with the represented numerosity (Figures 7–8) [32], [36]. If judging numerosity followed the predictions of feature detection and probability summation, which is hypothesized to be linked to the approximate number system, the coefficient of variation plotted as a function of element number would approach a log-log slope of −0.5 following the properties of a binomial distribution [32].

In Experiment 1, variation coefficients were modulated by viewing eccentricity in two ways. They increased in magnitude and increased in slope as a function of element number (Figure 7). Log-log slopes were −0.26, −0.39, −0.48, −0.44 and −0.54 at 2.25, 4.50, 6.75, 9.00 and 11.25 deg eccentricities, respectively. These data show that with increasing eccentricity, variation coefficients approached −0.5, the predicted slope from probability summation, a feature of the approximate number system [32].

In Experiment 2, variation coefficients had log-log slopes of −0.09, −0.04, −0.09, −0.02 and −0.15 as a function of element number at 2.25, 4.50, 6.75, 9.00 and 11.25 deg eccentricities, respectively. These slopes were much shallower, and show less modulation by viewing eccentricity than those from Experiment 1 (Figure 7). The shallow slopes of these variation coefficients were similar to slopes obtained for high visible gratings [32]. Notably, the magnitudes of the variation coefficient also did not vary with viewing eccentricity.

## Discussion

Collectively, these data suggest that (1) subitizing capacity is robust to the effects of viewing eccentricity, and (2) increasing eccentricity decreases enumeration accuracy and precision, but that (3) scaling stimulus size with eccentricity recovers the loss in enumeration performance.

### Enumeration processes

Notably, the effects of viewing eccentricity on enumeration functions of unscaled stimuli were very similar to the effects of luminance contrast [32]. As was previously found, key characteristics of enumeration functions were largely independent of contrast: subitizing capacities were between 3–4 items, while the log-log slopes of variation coefficients were negative, near −0.5. Because we detected empirical discontinuities in accuracies between small and large numbers, our results are consistent with the dichotomy between the precise and approximate number systems in enumeration functions. In contradistinction, negative slopes that become steeper with viewing eccentricity in the variation coefficients indicates that enumeration functions followed the predictions of probability summation (see Appendix B, [32]), and consistent with the idea that enumeration of small and large numerosities follow a continuous function. Together, the seemingly contradictory results from the analyses from the same data set suggest that the precise representation of numerosity supplements the approximate representation of numerosity: Precise number system operate over small numerosities, while the approximate number system operates spans both small and large numerosities [21], [32], [37]. The layered relationship between precise and approximate number systems is parsimonious with the recent evidence that attention modulates numerosity judgments within the subitizing range [26], [27], [28], [29], [30], [31]. Large attentional loads and high perceptual difficulty [32] reveal the extent of the approximate number system to small numerosities in typical adults.

Human neuroimaging work using fMRI and electrophysiological data in non-human primates has also suggested overlapping cortical mechanisms for enumerating small and large numbers. Attentional load has been found to modulate the cortical activity to small numbers in the right temporoparietal junction (rTPJ) [38]. In non-human primates, similar variability signatures for discriminating small and large numerosities have been reported in single-cell recordings in monkey prefrontal cortex and the intraparietal sulcus [39], which is consistent with monkey behavior [40].

### Other tasks and eccentricity

While this study is the first to have a comprehensive evaluation of subitizing capacity, response accuracy and response precision in visual enumeration as a function of eccentricity, our results match the conclusions of Parth and Rentschler [34], which had used briefly presented linear arrays of dots. They reported that a size scaling using a cortical magnification factor accounts for enumeration errors in the periphery when a length cue is available to observers, but not when it is unavailable from flanking bars. In the current study, we did not use distractors in order to control for confounding cues from luminance and length, but we used grating stimuli arranged in a circular array. Thus together with Parth and Rentschler's data [34], our results suggest that presence of distractors disrupts size scaling in the periphery, not the absence of length cues per se.

Along with other studies, we show that processing of multiple elements in the visual periphery is not qualitatively different from the visual fovea, with proper scaling of size and separation. Reading, the ultimate functional demonstration of integration of multiple elements, has been shown to be at a constant rate with eccentricity when the span of visual crowding was taken into account [13]; see also [11]. With cortical magnification scaling, the eccentricity effect in visual search has also been reported to disappear [41].

### Relationship to atypical vision

While the current study characterizes enumeration in the normal periphery, our results indicate that enumeration functions strabismic vision may qualitatively differ [42]. In Experiment 1, we show that the proportion correct at 1 element decreased as a function of eccentricity (Figure 2), but subitizing capacity was unaffected by eccentricity. However in strabismics, proportion correct at 1 elements was reported to be unaffected by strabismus, while subitization capacity decreases with strabismus (see Figure 2 in [42]). It remains to be tested whether the qualitative differences between the enumeration functions under conditions of normal eccentric viewing and strabismic vision are due to differences in stimulus configuration (i.e. grid [42] vs. ring [32]). Amblyopia associated with strabismus mainly affects foveal vision [43] and seems to be an unlikely candidate for this difference. Rather, these differences in enumeration functions might be due to cortical abnormalities that developmentally arise from atypical visuospatial experience in strabismic observers. It would be interesting to evaluate visual enumeration abilities in age-related macular degeneration, to determine how adult-onset retinal damage (and plasticity involved in the development of a preferred retinal locus) might affect apprehension of multiple elements. It is possible that the perception of numerosity, particularly subitization, might be compromised in atypical vision.

In summary, the objective of this study was to assess how parametrically varying viewing eccentricity affects visual enumeration. We found that subitizing capacity was generally unaffected viewing eccentricity while variation coefficients had steeper negative slopes with increasing viewing eccentricity. These results confirm the separate representation of precise and approximate number systems. However, they also support the notion that while the precise number system is restricted to small numerosities (represented by the subitizing limit) the approximate number system extends across both small and large numerosities (indexed by coefficients of variation) at large eccentricities. We also found that scaling the size of the targets neutralized the inaccuracies and imprecision associated with viewing eccentricity, suggesting that spatial resolution is the limiting factor in enumeration in the periphery.
